# A Comparative Study of Intramuscular Alfaxalone- or Ketamine-Based Anesthetic Mixtures in Gray Squirrels Undergoing Gonadectomy: Clinical and Physiologic Findings

**DOI:** 10.3390/ani10081402

**Published:** 2020-08-12

**Authors:** Sara Nannarone, Giulia Moretti, Federica Bellocchi, Laura Menchetti, Antonello Bufalari

**Affiliations:** Department of Veterinary Medicine, Perugia University, 06126 Perugia, Italy; giulia.moretti@studenti.unipg.it (G.M.); federica.bellocchi@studenti.unipg.it (F.B.); antonello.bufalari@unipg.it (A.B.)

**Keywords:** alfaxalone, anesthesia, dexmedetomidine, gonadectomy, ketamine, midazolam, *Sciurus carolinensis*, sevoflurane, squirrel

## Abstract

**Simple Summary:**

The loss of biodiversity is continuing at an alarming rate worldwide and continuous efforts are required to conserve and protect species and habitats. In 2011, Europe adopted the EU Biodiversity Strategy, aiming to halt the loss of biodiversity and ecosystem services. The increasing number of alien species is one of the leading environmental emergencies. The gray squirrel, introduced to Europe from the USA, is one of the most common and feared invasive species. The introduction of this non-native species is endangering the survival of the smaller native squirrel, called the “common European” or “red” squirrel. The gray squirrel has a devastating impact on native populations of red squirrels as it outcompetes them for food and territory, in addition to carrying diseases. Sterilization is an indirect and humane method for the eradication of gray squirrels; however anesthetic protocols have not yet been standardized. This study describes two anesthetic protocols used to induce chemical immobilization for safe handling, in order to perform the gonadectomy, simultaneously allowing a fast recovery, followed by re-introduction in urban parks. The two anesthetic protocols included a combination of dexmedetomidine and midazolam with either alfaxalone or ketamine. Comparisons of their efficacy and evaluations of their physiologic effects are reported.

**Abstract:**

The gray squirrel is one of the most common invasive species in Europe, whose presence is dangerous for the survival of the European red squirrel. To cope with this biological invasion and to safeguard biodiversity, the LIFE+U-SAVEREDS project aims to protect the red squirrel, by limiting the growth of the current population of gray squirrels and simultaneously promoting their eradication with surgical sterilization. This study compares two different anesthetic protocols, including dexmedetomidine (40 µg/kg) and midazolam (0.3 mg/kg) associated with ketamine (15 mg/kg; *n* = 25 squirrels) or alfaxalone (5 mg/kg; *n* = 22 squirrels). A blinded investigator evaluated the quality and onset of sedation, intraoperative anesthesia, and recovery, as well as the physiologic parameters for each animal. Alfaxalone provided a good quality of anesthesia with limited cardiovascular effects (*p* < 0.05) and good intraoperative myorelaxation. Ketamine induced complete relaxation in a shorter time (*p* < 0.05) and a rapid (*p* < 0.001) and excellent (*p* < 0.05) recovery. Despite the overall superiority of ketamine, alfaxalone appeared to be an adequate alternative anesthetic drug that can be administered without requiring intravascular access. It should be rapidly metabolized and excreted; however, it requires the combination of longer acting sedatives/myorelaxants to prevent a poor recovery quality.

## 1. Introduction

Invasive Alien Species (IAS) include animals and plants that have been introduced into an area where they do not normally exist. Generally, IAS have been introduced intentionally or unintentionally by human activities, and they are potentially harmful for the environment, economy, and human health. Outside of their native environment, IAS manage to stabilize, expand, and self-sustain their populations over time [1]. Nowadays, their introduction is one of the main factors of biodiversity loss on a global scale, second only to the destruction and fragmentation of habitats. IAS can have negative impacts on local biodiversity, and productive sectors (agriculture, industry, and fisheries), infrastructure, and public health [2,3].

The introduction and expansion of allochthonous species is a growing phenomenon that has gradually increased in recent years due to globalization, with the movement of greater volumes of goods and people. The introduction of about 12,000 allochthonous species (mostly consisting of terrestrial plants) is estimated to have occurred in Europe. Of these allochthonous species, 10–15% are potentially dangerous for biodiversity and about 3000 have been counted in Italy [4].

To protect native biodiversity, the EU has provided legislation against IAS with the EU regulation No. 1143/2014, containing “provisions aimed at preventing and managing the introduction and spread of invasive alien species”. This regulation has also been implemented in Italy with the specific DL No. 230/2017, and further by the Regions, Autonomous Provinces and National Parks with the support of the Higher Institute for Environmental Protection and Research (ISPRA), under the supervision of the Ministry of the Environment and Territorial Protection. The novelty of the aforementioned EU Regulation was the aim to establish a list of invasive species “of Union interest”. EU member states should prevent the introduction of invasive species, and provide early warnings and a rapid response system, including the immediate identification of newly established IAS nuclei, followed by their control or eradication.

The above mentioned list also includes the American gray squirrel, *Sciurus carolinensis*, whose introduction represents one of the best known and studied cases of biological invasion in Europe [5]. The alien species (gray squirrel in the current study) will be inclined to replace the native (red squirrel in the current study), via a complex process known as ‘competitive exclusion’ [6,7]. The two species occupy a similar ecological niche as they are both arboreal and diurnal, use the same natural resources, and produce a similar number of offspring during the same seasons. Inevitably, this overlap will allow the stronger species (gray squirrel) to overcome the weaker one (red squirrel), causing its extinction.

Well-documented examples of competitive exclusion (gray squirrel vs. red squirrel) are those reported in the UK and northern Italy [8,9,10,11,12,13]. Different susceptibilities to infections between the two species may rapidly amplify the competitive exclusion. As an example, the gray squirrel is a known vector for the Squirrelpox virus that caused a fatal outbreak of Squirrelpox among the United Kingdom’s red squirrel population. The infection has been reported to increase the replacement pace up to 25 times [14].

The LIFE+U-SAVEREDS project included an information campaign for citizens to raise awareness of the problem and the need to eradicate the gray squirrel from urban areas, without ignoring the public opinion, as the allochthonous species has acquired the sympathies of local people. The whole project included both a “direct” and “indirect” technique to remove alien squirrels: Capture by non-lethal traps followed by euthanasia, in line with animal welfare regulations, or surgical sterilization and release in pre-established city parks. Our institution has been involved in surgical sterilization, which has been demonstrated to be an important tool in management strategies aimed at removing critical populations of allochthonous squirrels from urban areas [13]. However, studies on the clinical and physiologic effects of anesthetics in wild squirrels are lacking in veterinary literature.

This study aimed to compare two different anesthetic protocols, including dexmedetomidine (40 µg/kg) and midazolam (0.3 mg/kg) associated with ketamine (15 mg/kg) or alfaxalone (5 mg/kg) administered intramuscularly (IM), in order to allow gonadectomy of male and female gray squirrels. We hypothesized that both techniques would provide similar intra-operative and post-operative conditions for the variables studied. Differences in the quality of anesthesia between the two protocols were assessed from both a clinical and physiological perspective.

## 2. Materials and Methods

The Veterinary Teaching Hospital (VTH) of the Department of Veterinary Medicine of the University of Perugia (Perugia, Italy) has been commissioned by the OIKOS Institute S.r.l. (protocol n. 18/2016) to manage the surgical sterilization of gray squirrels according to the project LIFE13 BIO/IT/204 U-SAVEREDS “*Management of grey squirrel in Umbria: conservation of red squirrel and preventing loss of biodiversity in Apennines*”. The ISPRA was the coordinating beneficiary of the LIFE13 BIO/IT/204 U-SAVEREDS Project, while the Region of Umbria, the Experimental Zooprophylactic Institute of Umbria and Marche, Legambiente Umbria, OIKOS Institute S.r.l., the Municipality of Perugia, and the Lazio Region were the associated beneficiaries. All procedures complied with Italian laws and followed indications reported in the Directive 2010/63/EU. The manuscript conforms with the CONSORT/SPIRIT statement.

### 2.1. Animals

From March 2017 to early June 2018, 68 gray squirrels were referred to the VTH to be included in the indirect eradication technique section of the project, involving surgical gonadectomy. The squirrels were trapped with collapsible Tomahawk live traps (Model 202.5, Tomahawk Live Trap Co., Hazelhurst, WI, USA) baited with hazelnuts and walnuts and located in urban areas populated by gray squirrels the night before or the morning prior to the planned day of the surgical procedure. The squirrels were captured in compliance with regulations regarding wildlife control explained in Article 19 of the Italian Law 157/92: “Rules for the protection of wild animals and homeotherms and for hunting”. Procedures were scheduled one day per week and only up to four animals underwent surgery on each study day, in order to allow the best perioperative management for each animal and the collection of all clinical parameters as defined in the protocol, including post-anesthetic recovery.

### 2.2. Procedures

#### 2.2.1. Animal Preparation

Squirrels were transferred to the VTH inside the live trap within 12 h from their capture. The day of capture was reported on the monitoring record of each animal.

**Figure 1:**
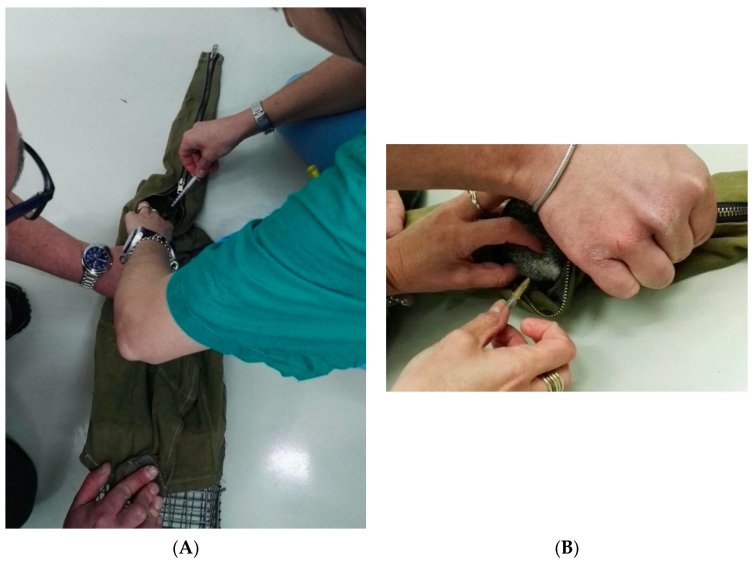
A gray squirrel moved from the live trap to the “capture-sleeve” to allow safe handling (**A**) and exteriorization of a hind limb for intramuscular injection through the zipper (**B**).

Squirrels were first weighted with an electronic scale (Mini Digital Platform Scale i2000, Shanghai China). This enabled the administration of proper anesthetic dosing based on effective, rather than estimated, body weight. To administer the intramuscular (IM) injection, animals were moved from the trap to a homemade “capture-sleeve” (conical piece of strong fabric, held closed with a zipper), which allowed safe handling of the squirrel and an efficient restraint facilitating exteriorization of the hindlimb (Figure 1).

The age of the animals in the study was defined as young or adult. The age assessment was based on the color of genitals and nipples, tail molt pattern and pigmentation, hair coverage, color and dimension of the vulva, and position of the testicles [15].

Squirrels were randomly allocated to receive one of two protocols, which differed in terms of the injectable anesthetic used: Ketamine (Ketavet 100^®^, MSD Animal Health S.r.l., Segrate, MI, Italy) or alfaxalone (Alfaxan^®^, 10 mg/mL, Dechra Veterinary Products S.r.l., Turin, TO, Italy). The RANDBETWEEN function of Excel was used for allocation. The same operator prepared and injected the anesthetic mixture in all animals IM. Injections were given in the gluteal or biceps femoris muscles with a 1 mL syringe and a 25 Gauge hypodermic needle. The quality and depth of anesthesia, as well as the quality of recovery, were assessed for each animal by using a Descriptive Scoring System (DSS) specifically developed for this study (see Table 1 below). Injection of the anesthetic mixture was defined as the starting point (T0). All animals were evaluated and scored according to the DSS (see Table 1) by an anesthetist (blinded to the type of anesthetic) at 3, 5, and 8 min. After 8 min, if the depth of anesthesia was still scored as 1 or 2, an additional half dose of the initial anesthetic mixture was administered IM.

To define the anesthetic protocols (molecules and dosages), a two-step pilot study of 21 squirrels was conducted by using the DSS. The DSS allowed the assessment of the anesthetic depth, including the evaluation of myorelaxation, scored from absent to excellent (1–3), according to an animal’s reaction after limb manipulation. This assessment also contributed to defining the anesthetic depth from inadequate to profound (1–3) (Table 1).

#### 2.2.2. Pilot Studies to Define the Final Anesthetic Mixtures and Final Anesthetic Protocol

The first pilot study involved 10 animals, randomized to receive an IM mixture of 30 µg/kg dexmedetomidine (Dextroquillan^®^, 0.5 mg/mL, A.T.I. S.r.l., Ozzano dell’Emilia, BO, Italy) with 15 mg/kg of ketamine (*n* = 5) or 4 mg/kg of alfaxalone (*n* = 5) (Table S1). About 60% of the animals in both groups, according to the DSS, were not adequately sedated after 8 min and required a second dose (Table S2). The first pilot study allowed us to understand that an increase of the dose of the dexmedetomidine (to 35 µg/kg) was needed and appropriate modifications were made for the second pilot study.

The second pilot study involved 11 squirrels (Table S1) randomized to receive an IM injection of the same (as in the first pilot study) ketamine dose (*n* = 5) or the same (as in the first pilot study) alfaxalone dose (*n* = 6) combined with 35 µg/kg of dexmedetomidine. Since more than 50% of the animals in both groups in the second pilot study did not produce satisfying results according to DSS (Table S2), the drug dosages were increased and a myorelaxant was added to improve the effects of the anesthetic mixture.

**Figure 2:**
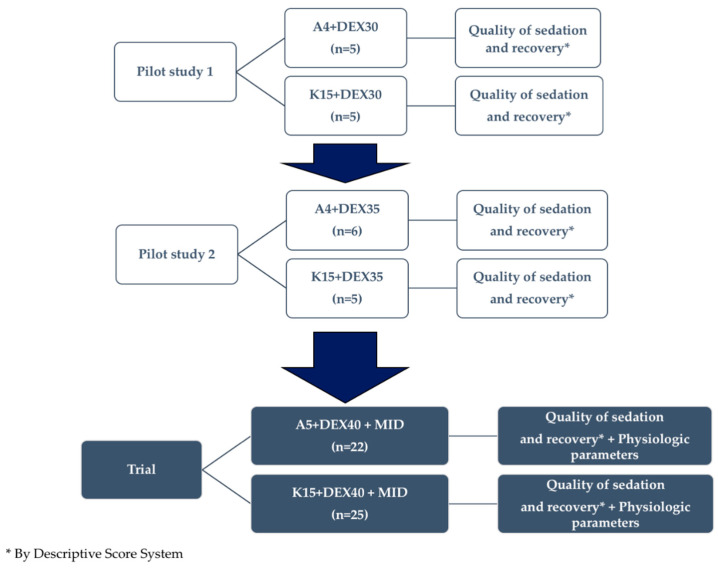
Experimental protocol that includes the two pilot studies. A4 = alfaxalone 4 mg/kg; DEX30 = dexmedetomidine 30 µg/kg; DEX35 = dexmedetomidine 35 µg/kg; K15 = ketamine 15 mg/kg; A5 = alfaxalone 5 µg/kg; DEX40 = dexmedetomidine 40 µg/kg; MID = midazolam.

The refined study protocols for the final trial of 47 squirrels included 0.3 mg/kg midazolam (Midazolam I.B.I., 0.5%^®^, Aprilia, LT, Italy), 40 µg/kg dexmedetomidine, and either 15 mg/kg ketamine (group K, *n* = 25) or 5 mg/kg alfaxalone (group A, *n* = 22) IM (Figure 2).

#### 2.2.3. Surgical and Anesthetic Procedure

**Figure 3:**
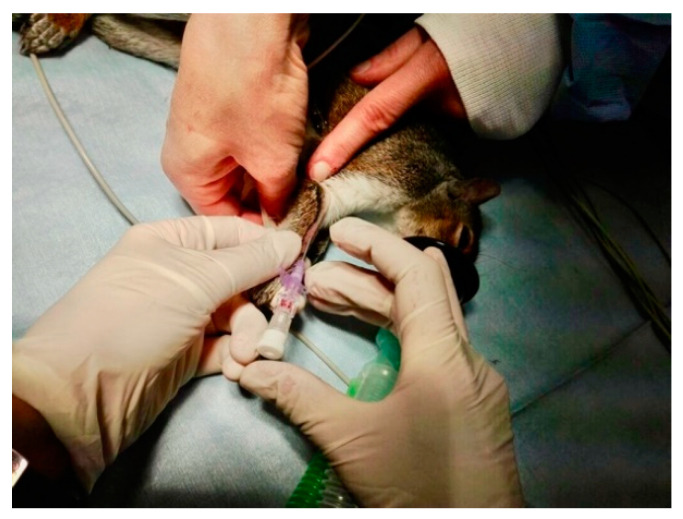
Aseptical catheterization of a cephalic vein in a gray squirrel under anesthesia receiving oxygen by a mask.

When safe handling was possible, animals were removed from the capture-sleeve and connected through a facemask to a Jackson-Rees non-rebreathing circuit. The oxygen flow was set at 300–500 mL/kg/min. If an inadequate level of anesthesia was noted during surgery, sevoflurane (SevoFlo^®^, Zoetis Italia S.r.l., Milano, MI, Italy) was administered to effect. Squirrels were placed on the surgical table on a temperature-regulated heating pad; their chest and upper limbs were further draped with bubble wrap packaging material to limit hypothermia. An attempt to aseptically catheterize a cephalic vein (Delta Ven^®^ 26 G, Delta Med S.p.A., Viadana, MN, Italy) was always made after clipping and scrubbing of the area and skin cut down, and was recorded for each animal, regardless of the outcome (Figure 3).

**Figure 4:**
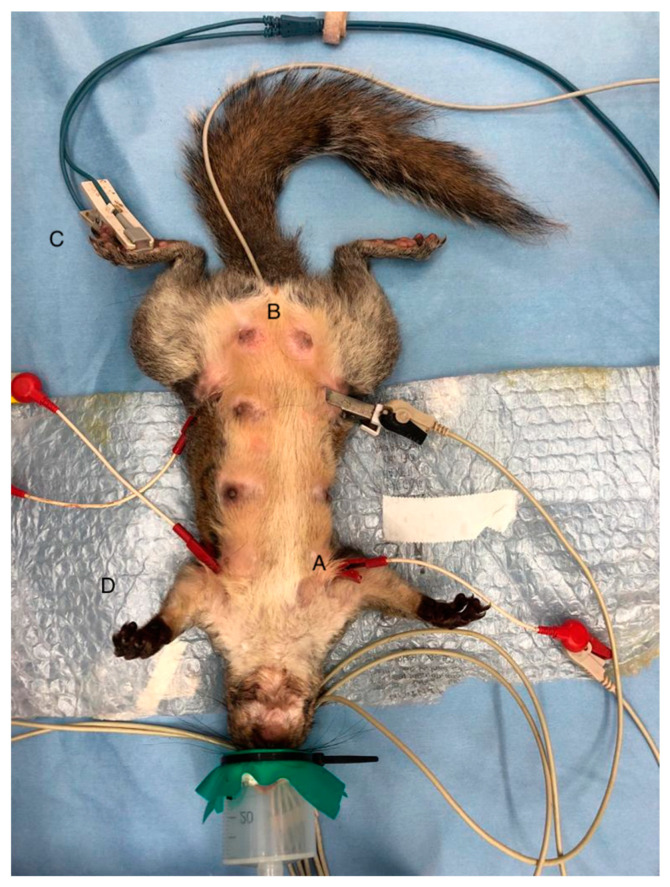
Female gray squirrel placed on the surgical table instrumented with monitoring for vital parameters such as the electrocardiogram (**A**), rectal temperature probe (**B**), and pulse oximeter (**C**), wrapped with a bubble wrap packaging material (**D**).

From the time of oxygen administration, physiologic parameters were collected and defined as baseline values. The electrocardiogram, heart rate (HR, beats/min), hemoglobin oxygen saturation (SpO_2_, %), and rectal temperature (T, °C) were continuously monitored (GT9003E, Guoteng Science & Technology Development Co., Ltd. Zhuhai, China) and recorded every 5 min, along with the respiratory rate (RR, breaths/min), which was assessed from movements of the reservoir bag (Figure 4).

All animals received 10 mg/kg enrofloxacin (Valemas 5^®^, Fatro S.p.A., Ozzano dell’Emilia, BO, Italy) diluted in 25 mL/kg Ringer Lactate (Ringer Lactate ^®,^ S.A.L.F. S.p.A. Laboratorio Farmacologico, Cenate Sotto, BG, Italy) subcutaneously.

The squirrels were placed in dorsal recumbency and a large area of the abdomen from the xiphoid to the inguinal area, or the scrotal area in mature males, was clipped, aseptically prepared for surgery with povidone iodine solution, and draped with a sterile adhesive incision film (Medincision op drape^®^, Mediberg, Calcinate, BG, Italy). Two types of surgical access were performed: Post-pubertal adult males, presenting external gonads, underwent median scrotal access, while females and pre-pubertal males, presenting intra-abdominal gonads, underwent median ventral celiotomy. All females underwent ovariectomy, but those presenting a pregnant uterus underwent ovariohysterectomy. Moreover, two surgical techniques were performed: Traditional gonadectomy by means of 3/0 polydioxanone absorbable sutures and scissor dissection, or gonadectomy through the bipolar vessel sealing device (Covidien Ligasure small jaw open instrument: 16.5 mm–19 cm Ligasure^TM^, Medtronic Italia S.p.A., Ancona, AN, Italy).

**Figure 5:**
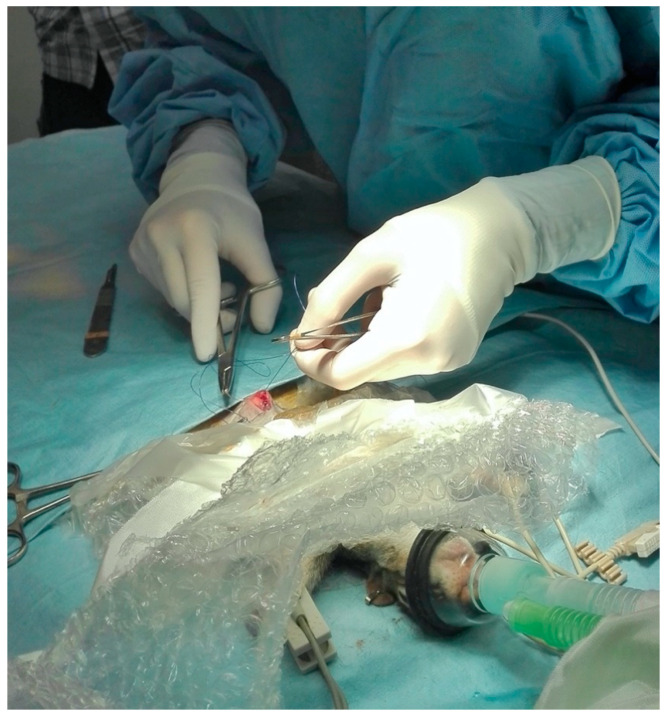
Surgical wound closure in a gray squirrel undergoing gonadectomy.

The laparotomic surgical access was sutured in three-layers and an intradermal suture pattern was performed (3/0 PDX, vetSuture^®^, Noévia S.A.S., Paris, France) in all animals (Figure 5), which was infiltrated with 4 mg/kg lidocaine (Lidocaina 2%^®^, ESTEVE S.p.A., Milan, MI, Italy) to provide local analgesia. At the end of surgery, 200 µg/kg atipamezole (Atipam^®^, Dechra Veterinary Products S.r.l., Turin, TO, Italy) and 1 mg/kg meloxicam (Metacam 2%^®^, Boehringer Ingelheim Italia S.p.A, Milan, MI, Italy) were administered IM.

**Figure 6:**
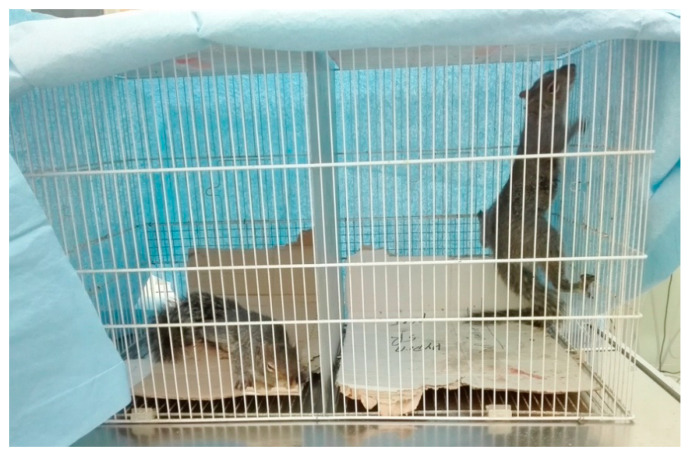
Two gray squirrels placed into a wire-mesh cage with a cardboard padded floor covered with a towel during recovery from anesthesia. A metal separator divided it into two spaces, allowing independent recovery. A still recumbent squirrel is present in the left section of the cage, while a squirrel displaying climbing activity (indicative of an excellent recovery), is noted in the right section of the cage.

During recovery, the squirrels were placed in a wire-mesh cage covered with a towel, with a cardboard padded floor and available food (Figure 6). The recovery time and quality were scored for each squirrel (Table 1). Squirrels were left in a warm undisturbed room overnight and released the following day if good vitality was present.

#### 2.2.4. Statistical Analysis

Only descriptive statistics were used to present the results of the two pilot studies (Tables S1 and S2).

Diagnostic graphics and Leven tests were used to check assumptions and outliers.

The associations between groups and sex or age were evaluated by a Chi square test. Body weight and basal physiologic values were compared between groups using independent sample t-tests. Results were presented as means and standard deviations (SD) or numbers and percentages.

Categorical variables related to the quality of sedation were analyzed by Generalized Linear Models (GLM) using binomial or multinomial as probability distributions and logit or cumulative logit as link functions. Z-tests were used to compare column proportions. Continuous variables were analyzed by GLM, setting normal and identity as the probability distribution and link function, respectively. Pairwise comparisons were performed using the least significant difference. These results were expressed as estimated marginal means ± standard error (SE). The models evaluated the main effects of the group (2 levels: K and A), sex (2 levels: Males and female), and capture day (2 levels: 0 and 1, day before and same day of surgery, respectively). For repeated measurements, the time (minutes from connection to the breathing circuit, from 0 to 30 min) and the baseline values were included as covariates.

Statistical analyses were performed with SPSS Statistics version 23 (IBM, SPSS Inc., Chicago, IL, USA). Statistical significance occurred when *p* ≤ 0.05.

## 3. Results

The two groups were homogeneous in terms of the demographic characteristics of animals: No differences were found for gender (*p* = 1.000), body weight (*p* = 0.759), age (*p* = 0.755; Table 2), and basal physiologic values (all *p* > 0.05).

**Figure 7:**
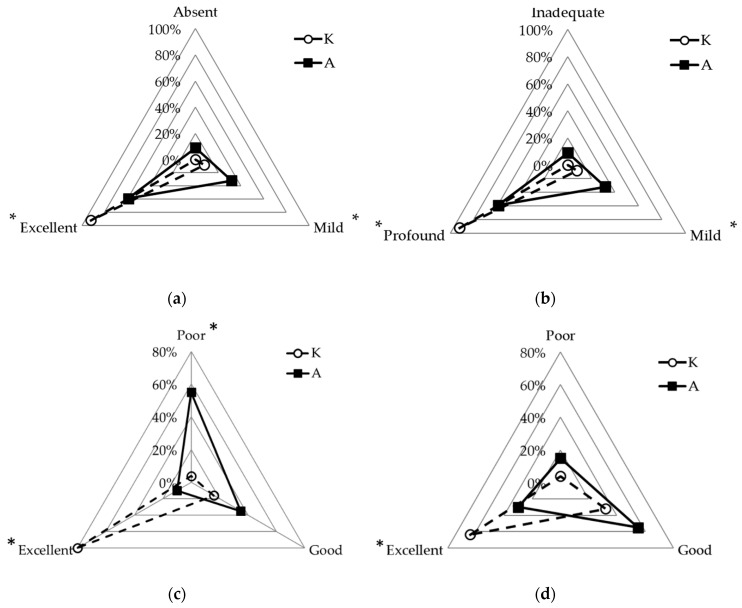
Radar charts for muscle relaxation (**a**), depth of anesthesia (**b**), recovery time (**c**), and quality (**d**) for wild gray squirrels undergoing gonadectomy, randomized to receive two anesthetic mixtures IM: Ketamine-dexmedetomidine-midazolam (group K, *n* = 25) and alfaxalone-dexmedetomidine-midazolam (group A, *n* = 22). Asterisks indicate the levels of each variable in which the differences between groups were significant at the 0.05 level.

The quality of anesthesia, in terms of myorelaxation and depth, was significantly different between groups. Muscle relaxation was scored as “excellent” in 92.0% (*n* = 23) and 59.1% (*n* = 13), “mild” in 8.0% (*n* = 2) and 31.8% (*n* = 7), and “absent” in 0.0% (*n* = 0) and 9.1% (*n* = 2) of squirrels in groups K and A, respectively (*p* = 0.011; Figure 7a). The same percentages were recorded for the parameter depth of anesthesia (*p* = 0.011; Figure 7b). The gender and day of capture did not affect the quality of anesthesia.

The recovery time was significantly different between groups as it was scored as “poor” in 4% and 55% and “excellent” in 80% and 10% of squirrels in groups K and A, respectively (*p* < 0.001; Figure 7c). The recovery quality was “excellent” in 64% and 30%, “good” in 32% and 55%, and “poor” in 4% and 15% of squirrels in groups K and A, respectively (*p* = 0.016; Figure 7d). The gender and day of capture had no influence on the recovery outcome.

The time for safe manipulation from T0 was lower in group K (5 ± 2 min) than in group A (9 ± 4 min; *p* < 0.001) and it was not influenced by gender (*p* = 0.609) or by the day of capture (*p* = 0.750). The intravenous catheter was successfully placed in 48% and 45% of squirrels in groups K and A, respectively.

Table 3 presents timescales relating to the procedure. Over a total anesthetic time of 34 ± 2 and 31 ± 2 min in group K and A, respectively, sevoflurane was administered for a mean time of 2 ± 1 and 3 ± 1 min in group K and A, respectively. The median (range) vaporizer setting was 3.5% (2–4.5) and 3.5% (3–5) in group K and A, respectively. The surgery time was not affected by the group, but it was higher in females (38 ± 2 min) than males (28 ± 2 min; *p* < 0.001). The surgery time was further investigated by including the laparotomy factor in the GLM. The model showed that, in addition to the gender, the laparotomy closely approached statistical significance (11.3 ± 1.1 min and 14.6 ± 0.9 min in the absence and presence of laparotomy, respectively; *p* = 0.053).

The times required to achieve sternal recumbency, to stand up, and to finally climb the side of the cage after atipamezole administration were significantly shorter in group K when compared to group A (*p* < 0.001), regardless of gender and the day of capture (Table 3).

Respiratory depression, in terms of transient apnea, was recorded in 7/25 (29.2%) and 6/22 squirrels (28.6%; *p* = 0.965); cardiac arrhythmia, referred to as second-degree atrio-ventricular block or ventricular premature contractions, was recorded in 2/25 (8.3%) and 1/22 squirrels (4.8%; *p* = 0.636); and hypothermia (defined as a rectal temperature <36.5 °C for more than 10 min) was recorded in 9/25 (37.5%) and 8/22 squirrels (38.1%; *p* = 0.967) in group K and A, respectively.

Table 4 shows the results of the physiologic parameters continuously monitored throughout the procedure. The heart rate decreased over time with respect to the baseline (*p* < 0.001) of about 14% in both groups. It was significantly influenced by the day of capture (*p* = 0.043); in particular, there was a decrease of about 16% and 12.5% when animals were trapped the day before and the same day of surgery, respectively. The respiratory rate decreased compared to the baseline only in group A (*p* < 0.001). The rectal temperature decreased over time (*p* < 0.001) with respect to the baseline and reached significantly lower values in group K when compared to group A (*p* = 0.037).

Regardless of the group of study, we further evaluated the effect of season on the basal physiologic parameters of wild gray squirrels undergoing gonadectomy. Surprisingly, HR was higher during fall (273 ± 10 beats/min; *p* = 0.022) compared to winter (242 ± 12 beats/min) and summer (225 ± 13 beats/min; Table S3).

The overall mortality rate was 5.8%: Pilot study 1, group K15+DEX30 (*n* = 1); final trial, group K (*n* = 2); group A (*n* = 1).

## 4. Discussion

This is the first study reporting the clinical and physiologic effects on gray squirrels anesthetized with two different anesthetic protocols during surgical gonadectomy for population control.

A similar campaign of IAS population control was conducted by Scapin et al. [13] and included an IM association of dexmedetomidine (20 µg/kg) and ketamine (15–20 mg/kg), followed by inhalation anesthesia with 1.5% isoflurane in oxygen. However, in that study, anesthetics were administered according to an estimated body weight of 500 g, which was often overestimated. In addition, these authors did not monitor any physiologic parameters and a qualitative description of the adopted anesthetic protocol was not described [13].

Anesthesia or sedation for wildlife casualties is often required for surveying and monitoring purposes, i.e., routine examination, weighing, blood or tissue sampling, and the implantation of microchips. Trapping and handling is stressful for wild animals and may result in injuries [16,17]. Chemical immobilization may include inhalant anesthetics, which necessarily require costly instruments that may be cumbersome in a field condition and can determine cardiovascular depression [18], or injectable drugs. A suitable injectable anesthetic combination should involve short-acting anesthetic agents with a wide safety margin and that are preferably reversible. Usually, injectable anesthesia in rodents is performed by combining dissociative drugs such as ketamine with α2-receptor agonists. Unfortunately, these protocols produce prolonged sedation, and ketamine-based combinations have been feared to cause protracted recovery [19,20,21]. Therefore, the present study aimed to evaluate two anesthetic protocols using multiple parameters, in order to define the one that best protects the animal’s well-being.

The two protocols included dexmedetomidine (40 µg/kg) and midazolam (0.3 mg/kg) associated with ketamine (15 mg/kg; group K) or alfaxalone (5 mg/kg; group A), eventually followed by sevoflurane in oxygen. The final dose of the selected drugs was due to the outcome of two pilot studies according to a DSS defined to evaluate the quality of myorelaxation and anesthesia achieved. Sevoflurane has not been extensively evaluated for use on wild mammals and it was required for a very limited time over the entire procedure in some animals in the study, demonstrating its efficacy as a fast agent for deepening the anesthetic plan.

The combination of three injectable drugs was expected to cause a potential synergistic effect providing balanced surgical anesthesia in squirrels. Ketamine is a noncompetitive N-methyl-D-aspartate glutamate receptor antagonist that produces anesthesia by inducing a dissociation of sensory, motor, integrative, memory, and emotional activities in the brain, leading to catalepsy without central nervous system depression. However, it is a poor muscle relaxant and therefore, it is used in combination with other drugs, such as benzodiazepines or the α2-adrenergic agonist [22]. In this context, dexmedetomidine and midazolam were chosen, respectively, as the newest α2-adrenergic agonists with sedative and analgesic properties, and the benzodiazepine, γ-amino-butyric acid subtype A (GABA_A_) receptor agonist, with muscle relaxant properties, cardiovascular sparing effects, and predictable IM absorption [23].

Alfaxalone is a neuroactive steroid that acts as a positive allosteric modulator of GABA_A_ receptors, causing hyperpolarization of the neuron and producing a strong anesthetic effect [24]. It has a wide margin of safety and has been used as a general anesthetic in several species, including Wistar rats [25], dogs [26], cats [27], ferrets [28], and rabbits [29], although it lacks analgesic activity.

Both midazolam and alfaxalone are GABA_A_ receptor agonists, and there is a presumed additive effect when acting at a similar receptor during co-administration [30].

In our study, surgery and anesthesia times were similar to those reported by Scapin et al. [13], but we appreciated a significant influence of gender, irrespective of the group of study. Females required a surgical time that was 7 min longer than males and anesthesia was about 10 min significantly longer, accordingly. Preliminary results comparing Ligasure and traditional surgical techniques employed during gonadectomy in squirrels are reported elsewhere [31]; however, Ligasure application is desirable as it provides hemostasis by denaturing collagen and elastin of the vessel wall and surrounding connective tissue in a short time, lowering intraoperative complications and the anesthesia time.

The time for squirrels’ manipulation was significantly shorter in group K (about 5 min). The manipulation time is quite similar to that reported by Parker [32] (4.6 min) using isoflurane administered to 91 squirrels requiring blood and tissue samples in field conditions. Ketamine-based protocols produced better results based on the DSS, according to the quality and depth of anesthesia and recovery quality when compared to alfaxalone-based protocols. However, good DSS scores were also obtained with the use of alfaxalone. Alfaxalone is an injectable anesthetic recently marketed in veterinary medicine and is not a controlled substance. It could be used as an alternative to ketamine, which is a controlled drug.

To the best of the authors’ knowledge, this is the first study describing vital parameter variation during anesthesia in gray squirrels. A mean heart rate of about 259 beats/min has been reported in two captive gray squirrels; however, it may be lower in free-ranging squirrels. Moreover, a fear bradycardia of 25% was consistently recorded upon placing animals into the nest box after stimulation [33]. A mean rectal temperature of 36.4 °C (34–39.5 °C) has been reported in ketamine-anesthetized gray squirrels [34] and a mean of 38.7 °C (37.5–39.9 °C) in caged animals during the summer [35]. Parker et al. [32], during about 9.5 min of isoflurane immobilization, only recorded the body temperature, pulse rate, and respiratory rate once. They described significantly lower values of the rectal temperature (39.2 °C) and respiratory rate (116 breaths/min) during winter time captures, and a higher heart rate (373 beats/min) during summer time captures; similarly, the recovery time occurred within 2 min during summer captures and was statistically shorter than winter captures (4 min).

The decrease in rectal temperature is an expected side effect of general anesthesia [36] and this happened in our study, with a significant decrease below 37 °C in group K and a progressive reduction over time compared to the baseline in both groups. However, apparently, these values are still within acceptable ranges [34]. Small mammals can rapidly become hypothermic during anesthesia due to the large body surface area to volume ratio and this can become an important factor in the winter. We did not view any influence of seasonality on the degree of the recorded body temperature (Table S3), and devices were used to limit heat loss, such as thermal pads and insulating material, resulting in 36% of animals showing relevant hypothermia (<36.5 °C for more than 10 min) in each group.

Regardless of the group, there was a decrease in HR values when compared to the baseline, which was significantly evident over time and influenced by the capture day. This decrease in HR is not surprising, given that dexmedetomidine causes both centrally mediated bradycardia and reflexive bradycardia due to its vasoconstrictive action on α2-receptors [37]. Indeed, the recorded negative influence of trapping the day before surgery can confirm the fear bradycardia described in gray squirrels when a cover is available after a stress stimulus [33].

Regardless of the group, we reported an overall post-operative mortality rate of 5.8%, and our data were similar to what was reported by Scapin et al. [13], who described a 6.2% of death rate in animals that presented with a poor physical condition prior to capture and only during fall or winter time.

Since alfaxalone produces hypoventilation, oxygen support may be advisable; its anesthetic profile resembles that of propofol, and its effects may be potentiated when combined with sedatives and analgesics [38]. According to the latter statement, in our study, RR was significantly lower in group A, with mean values <90 breaths/min already at the baseline, which was recorded as soon as the animal was connected to the breathing circuit. However, SpO_2_ values remained >95% in both groups. This is similar to what was reported in Sprague Dawley rats receiving alfaxalone-based combinations. In these rats, ventilatory depression, determined by marked decreases in both RR and SpO_2_, was associated with both alfaxalone and the absence of oxygen supplementation [39]. All squirrels of our study received oxygen via a face mask, which may justify the lack of a SpO_2_ decrease.

The current study demonstrated that alfaxalone, in combination with dexmedetomidine and midazolam, can provide reliable anesthesia in squirrels. Nevertheless, the time required for complete relaxation was longer and less pronounced when compared to ketamine-based anesthesia, requiring a redosing of anesthetics in 18% of cases before safe manipulation. Moreover, after atipamezole administration, recovery was rapid and excellent in group K, but longer and worse in group A, as tremors, twitching, and rolling were observed in the majority of the squirrels anesthetized with alfaxalone-based protocols, similar to what described in chinchillas [40] and mice [41]. The latter undesired effects could be reduced by avoiding atipamezole administration, thus masking the neurosteroid side effects; however, a control group without antagonization of dexmedetomidine was not considered in our study.

In the present study, there could have been several sources of bias, such as the different genders and surgical approaches, the age and the variability of the physical condition of the animals, the season, and the day of capture. However, the groups were balanced for sex and age. Moreover, the inclusion of these factors as covariates in the statistical models increased the reliability of the results.

## 5. Conclusions

Balanced anesthesia takes advantage of the different beneficial effects of several drug classes. It aims to induce immobility, a loss of consciousness, muscle relaxation, and analgesia by using a combination of drugs with specific individual properties and to reduce the potential side effects associated with individual agents. Ketamine-based protocols produced better results in terms of the quality and depth of anesthesia and the recovery quality when compared to alfaxalone-based protocols. However, good DSS scores were also obtained when using alfaxalone.

In Europe, alfaxalone, unlike ketamine, is not a controlled substance. It has the potential to be a suitable anesthetic drug in wild exotic animals, because it can be administered without requiring intravascular access, it has limited cardiovascular effects, and it is rapidly metabolized and excreted, potentially resulting in rapid recovery [42]. However, it is the authors’ opinion that in squirrels which require recovery from anesthesia to be shorter than 2 h, alfaxalone should be avoided or the residual effects of longer acting sedatives/myorelaxants should be allowed to persist, i.e., no antagonization should be eventually provided, to limit a poor recovery quality.

## Figures and Tables

**Table 1:** Descriptive Scoring System (DSS) developed for this study and used to categorize the quality and depth of anesthesia and the quality of recovery in wild gray squirrels undergoing gonadectomy.

**Score**	**Myorelaxation**
1	Absent: Muscle tone in the pelvic and thoracic limbs, pedal reflex, head movement
2	Mild: Some muscle tone in thoracic limbs and pedal reflex
3	Excellent: No muscle tone, completely relaxed animal
**Score**	**Depth of Anesthesia**
1	Inadequate: Responsive to manipulation in the capture-sleeve (legs and paw pinch). Additional ½ dose is required
2	Mild: Purposeful response to manipulation (legs and paw pinch). Additional ½ dose is required
3	Profound: Relaxed and unresponsive to manipulation (legs and paw pinch).
**Score**	**Recovery Time**
1	Poor: Longer than 60 min from atipamezole
2	Good: From 10 to 60 min from atipamezole
3	Excellent: Within 10 min from atipamezole
**Score**	**Recovery Quality**
1	Poor: Long lasting lateral recumbency and diffuse tremors
2	Good: Lateral or sternal recumbency associated with head tossing and uncoordinated legs movements, minor tremors, or ataxia once standing
3	Excellent: Quickly standing and climbing, no tremors

**Table 2:** Demographic characteristics and baseline physiologic parameters of wild gray squirrels undergoing gonadectomy and receiving either ketamine-dexmedetomidine-midazolam (group K, *n* = 25) or alfaxalone-dexmedetomidine-midazolam (group A; *n* = 22) intramuscularly (IM).

Parameter		Group	
		K	A
Gender	Female (*n*, %)	13 (52.0%)	11 * (50.0%)
	Male (*n*, %)	12 (48.0%)	11 (50.0%)
Body weight (g; mean ± SD (range))		469 ± 73 (311–600)	436 ± 78 (245–579)
Age	Young	8 (32.0%)	8 (36.4%)
	Adult	17 (68.0%)	14 (63.6%)
HR (beats/min; mean ± SD)		264 ± 52	250 ± 40
RR (breaths/min; mean ± SD)		119 ± 55	82 ± 43
SpO_2_ (%; mean ± SD)		96 ± 6	98 ± 1
Temperature (°C; mean ± SD)		38 ± 1	38 ± 1

* Two pregnant squirrels; SD = standard deviation; HR = heart rate; RR = respiratory rate; SpO_2_ = hemoglobin oxygen saturation.

**Table 3:** Parameters of clinical conditions at arrival, additional anesthetics required according to a Descriptive Scoring System (DSS), procedural characteristics, and recovery times recorded for wild gray squirrels undergoing gonadectomy and randomized to receive two anesthetic mixtures IM: Ketamine-dexmedetomidine-midazolam (group K, *n* = 25) and alfaxalone-dexmedetomidine-midazolam (group A, *n* = 22). Values are numbers and percentages or estimated marginal means ± standard error.

Parameter		Group		Significance		
		K	A	Group	Gender	Capture Day ^#^
Pre-existing injuries	No	19 (76.0%)	21 (95.5%)	0.095	0.068	0.755
	Yes	6 (24.0%)	1 (4.5%)			
Additional anesthetics, 8 min from T0	No	25 (100.0%)	18 (81.8%)	0.999	0.683	0.999
	Yes	0 (0.0%)	4 (18.2%)			
Laparotomy	No	10 (40.0%)	9 (40.9%)	1.000	0.998	0.999
	Yes	15 (60.0%)	13 (59.1%)			
Ligasure	No	19 (76.0%)	15 (75.0%)	0.989	0.216	0.369
	Yes	6 (24.0%)	5 (25.0%)			
Sevoflurane administration (min tot)		2 ± 1	3 ± 1	0.176	0.331	0.839
Anesthesia duration (min)		34 ± 2	31 ± 2	0.278	<0.001 *	0.442
Surgery duration (min)		14 ± 1	13 ± 1	0.309	<0.001 ^†^	0.925
Time to achieve sternal recumbency (min from atipamezole)		17 ± 13	81 ± 13	<0.001	0.493	0.298
Time to stand up (min from atipamezole)		17 ± 9	96 ± 9	<0.001	0.482	0.693
Time to climb the cage (min from atipamezole)		21 ± 12	137 ± 14	<0.001	0.422	0.494

^#^ Capture day: Indicates whether animals were trapped the day before or the same day of the scheduled surgery. *p* values in bold denote statistical significance at the 0.05 level. * Estimated marginal means for the sex effect ± SE = 38 ± 2 min and 28 ± 2 min for females and males, respectively. ^†^ Estimated marginal means for the sex effect ± SE = 17 ± 1 min and 10 ± 1 min for females and males, respectively. T0 = Injection of the anesthetic mixture.

**Table 4:** Physiologic parameters monitored continuously in wild gray squirrels undergoing gonadectomy and randomized to receive two anesthetic mixtures IM: Ketamine-dexmedetomidine-midazolam (group K, *n* = 25) and alfaxalone-dexmedetomidine-midazolam (group A, *n* = 22). Values are estimated marginal means ± standard error.

Parameter	Group		Baseline Value *	Significance				
	K	A		Group	Gender	Capture Day ^#^	Baseline Value	Time
HR (beats/min)	219 ± 3	221 ± 3	256	0.646	0.272	0.043 ^†^	<0.001	<0.001
RR (breaths/min)	108 ± 4	84 ± 4	100	<0.001	0.274	0.793	<0.001	0.790
SpO_2_ (%)	98 ± 0	98 ± 0	97	0.932	0.400	0.935	0.422	0.669
Temp. (°C)	36.8 ± 0.1	37.1 ± 0.1	38.3	0.037	0.637	0.076	<0.001	<0.001

* As it appears in the model. ^#^ Capture day: indicates whether animals were trapped the day before or the same day of the scheduled surgery. ^†^ Estimated marginal means: 214 ± 5 and 224 ± 2 beats/min for trapping the day before and the same day of surgery, respectively. *p* values in bold denote statistical significance at the 0.05 level. HR = heart rate; RR = respiratory rate; SpO_2_ = hemoglobin oxygen saturation.

## Supplementary Materials

The following are available online at https://www.mdpi.com/2076-2615/10/8/1402/s1, Table S1: Demographic characteristics of wild gray squirrels undergoing gonadectomy during pilot study 1 and 2; Table S2: Parameters related to the quality of sedation, surgical techniques, and recovery phase for wild gray squirrels undergoing gonadectomy during pilot study 1 and 2; Table S3: Effect of the season on the basal physiologic parameters of wild gray squirrels undergoing gonadectomy.
