# Supplementary material for: A Comparative Study of Intramuscular Alfaxalone- or Ketamine-Based Anesthetic Mixtures in Gray Squirrels Undergoing Gonadectomy: Clinical and Physiologic Findings
Source: Animals (Basel). 2020 Aug 12;10(8):1402. doi: 10.3390/ani10081402 (PMC7460254; doi:10.3390/ani10081402)
Supplement: Supplementary file 1 [file animals-10-01402-s001.pdf]

Table S1. Demographic characteristics of wild grey squirrels undergoing gonadectomy during pilot study 1 and 2 randomized to receive two anesthetic mixtures IM including ketamine (15 mg/kg) or alfaxalone (4 mg/kg) with different dexmedetomidine doses: alfaxalone-dexmedetomidine 30 μg/kg (group A+30DEX; n=5), ketamine-dexmedetomidine 30 μg/kg (group K+30DEX; n=5), alfaxalone-dexmedetomidine 35 μg/kg (group A+35DEX; n=6) and ketamine-dexmedetomidine 35 μg/kg (group K+35DEX; n=5).

		**Pilot study 1 (n=10)**		**Pilot study 2 (n=11)**	
		**A+30DEX**	**K+30DEX**	**A+35DEX**	**K+35DEX**
Sex	Female (n, %)	2 (40.0%)	1 (20.0%)	2 (33.3%)	3* (60.0%)
	Male (n, %)	3 (60.0%)	4 (80.0%)	4 (66.7%)	2 (40.0%)
Body weight (g; mean± SD)		424±105	530±45	477±92	520±45
Age	Young	2 (40.0%)	1 (20.0%)	4 (66.7%)	1 (20.0%)
	Adult	3 (60.0%)	4 (80.0%)	2 (33.3%)	4 (80.0%)

*1 pregnant squirrel.

Table S2. Parameters related to the quality of sedation, surgical techniques and recovery phase on wild grey squirrels undergoing gonadectomy during pilot study 1 and 2 randomized to receive two anesthetic mixtures IM including ketamine (15 mg/kg) or alfaxalone (4 mg/kg) with different dexmedetomidine doses: alfaxalone-dexmedetomidine 30 μg/kg (group A+30DEX; n=5), ketamine-dexmedetomidine 30 μg/kg (group K+30DEX; n=5), alfaxalone-dexmedetomidine 35 μg/kg (group A+35DEX; n=6) and ketamine-dexmedetomidine 35 μg/kg (group K+35DEX; n=5). Descriptive statistics: values are numbers and percentages.

**Parameter**		**Pilot study 1 (n=10)**		**Pilot study 2 (n=11)**	
		**A+30DEX**	**K+30DEX**	**A+35DEX**	**K+35DEX**
**Muscle relaxation**	**Absent**	0 (0.0%)	0 (0.0%)	0 (0.0%)	0 (0.0%)
	**Mild**	5 (100.0%)	3 (60.0%)	3 (50.0%)	4 (80.0%)
	**Excellent**	0 (0.0%)	2 (40.0%)	3 (50.0%)	1 (20.0%)
**Depth of anesthesia**	**Inadequate**	0 (0.0%)	0 (0.0%)	0 (0.0%)	0 (0.0%)
	**Mild**	5 (100.0%)	3 (60.0%)	3 (50.0%)	4 (80.0%)
	**Profound**	0 (0.0%)	2 (40.0%)	3 (50.0%)	1 (20.0%)
**Recovery time**	**Poor**	0 (0.0%)	0 (0.0%)	2 (33.3%)	0 (0.0%)
	**Good**	4 (80.0%)	1 (20.0%)	1 (16.7%)	0 (0.0%)
	**Excellent**	1 (20.0%)	4 (80.0%)	3 (50.0%)	5 (100.0%)
**Recovery quality**	**Poor**	1 (20.0%)	0 (0.0%)	1 (16.7%)	0 (0.0%)
	**Good**	2 (40.0%)	0 (0.0%)	3 (50.0%)	1 (20.0%)
	**Excellent**	2 (40.0%)	5 (100.0%)	2 (33.3%)	4 (80.0%)

Table S3. Effect of the season on basal physiologic parameters of wild grey squirrels undergoing gonadectomy (estimated marginal means ± standard error).

	**Season**				***p* value**
	**Winter**	**Spring**	**Summer**	**Fall**	
**RR_basal**	102±11	100±13	121±14	89±11	0.338
**HR_basal**	242^{a}±12	244^{ab}±12	225^{a}±13	273^{b}±10	**0.022**
**SpO_2_basal**	98±1	98±1	97±1	96±1	0.587
**T_basal**	38±1	38±1	38±1	38±1	0.936

For each row, values followed by the same superscript letter do not differ significantly ($p<0.05$).
